# Combination Effects of Docetaxel and Doxorubicin in Hormone-Refractory Prostate Cancer Cells

**DOI:** 10.1155/2012/832059

**Published:** 2012-07-01

**Authors:** Eleftheria Tsakalozou, Allison M. Eckman, Younsoo Bae

**Affiliations:** Department of Pharmaceutical Sciences, College of Pharmacy, University of Kentucky, Lexington, KY 40536-0596, USA

## Abstract

Combination effects of docetaxel (DOC) and doxorubicin (DOX) were investigated in prostate cancer cells (PC3 and DU145). Combination indices (CIs) were determined using the unified theory in various concentrations and mixing ratios (synergy: CI < 0.9, additivity: 0.9 < CI < 1.1, and antagonism: CI > 1.1). DOC showed a biphasic cytotoxicity pattern with the half maximal inhibitory concentration (IC50) at the picomolar range for PC3 (0.598 nM) and DU145 (0.469 nM), following 72 h drug exposure. The IC50s of DOX were 908 nM and 343 nM for PC3 and DU145, respectively. Strong synergy was seen when PC3 was treated with DOC at concentrations lower than its IC50 values (0.125*~*0.5 nM) plus DOX (2*~*8 times IC50). Equipotent drug combination treatments (7 × 7) revealed that the DOC/DOX combination leads to high synergy and effective cell death only in a narrow concentration range in DU145. This study provides a convenient method to predict multiple drug combination effects by the estimated CI values as well as cell viability data. The proposed DOC/DOX mixing ratios can be used to design combination drug cocktails or delivery systems to improve chemotherapy for cancer patients.

## 1. Introduction

Chemotherapy is widely used to treat cancer patients, but most anticancer drugs show a narrow therapeutic window [1]. Recent drug discovery and development studies are focused on maximizing the therapeutic efficacy of potent anticancer drugs at low dose levels [2]. Targeted drug treatment and controlled drug delivery are techniques used widely to achieve effective cancer treatment with reduced toxicity. Targeted drug treatment is based on the rationale to use therapeutic agents that act on molecular targets over expressed in cancer cells, and thus to suppress cancer cell growth or induce cell death selectively [3, 4]. Controlled drug delivery is an approach to transport potent therapeutic agents to disease lesions at the right dose level and time [5]. In both approaches, cancer patients can benefit from improved therapeutic efficacy.

We have been developing nanoparticulate drug delivery systems that carry various therapeutic agents alone or in combination to tumor tissues [6]. Despite promising outcomes in preclinical and clinical studies, the therapeutic efficacy of drug delivery systems appears to heavily rely on the biological activity of drug payloads. Drug activity is often determined by the concentrations at the site of action and the delivery schedule. Drug mixing ratios also become critical when multiple drugs are used in combination [7]. Multiple drugs can compete with each other for the same transporter, molecular target, or have conflicting effects on the cell cycle. Studies suggest that cancer cells are sensitive to multiple drugs at a certain drug mixing ratio, and that that optimal mixing ratio must be retained in tumor tissues to achieve the maximal drug combination effect [8–12]. Therefore, it is critical to identify the optimal combination settings and study the cellular response in detail.

In this study, we report the combination effects of docetaxel (DOC) and doxorubicin (DOX), anticancer drugs used widely in clinical applications, on the cell viability of established human prostate cancer cell lines (PC3 and DU145) at various drug concentrations and mixing ratios. DOC is a semisynthetic taxane that inhibits the depolymerization of the microtubules and results in G2/M phase cell cycle arrest [13–16]. DOX is a member of the anthracyclines, which acts by intercalating with the DNA [17–19]. The latter results in cell death attributed to DNA cleavage mediated by topoisomerase II (TOP2) and is accompanied by G2/M phase cell cycle arrest [20]. PC3 and DU145 are used as *in vitro* models for the advanced hormone-refractory prostate cancer (HRPC) [21–23]. 

HRPC has a low therapeutic response to conventional chemotherapy [24–26]. DOC appeared to be the most potent cytotoxic agent used today for the treatment of HRPC [24, 27–29], yet the reported toxicity is still high. Extensive optimization of DOC regimens has taken place to reduce drug toxicity, yet the drug efficacy also became variable [30]. A number of clinical trials have shown that combination of DOC and anthracyclines would benefit HRPC patients by sensitizing cancer cells at lower DOC doses [13, 14, 31–36].

DOC has been concurrently used with other anticancer drugs, such as prednisone, mitoxantrone, and estramustine to treat prostate cancer [36–40]. Previous *in vitro* studies showed that pretreatment with DOC rendered cancer cells (BCap and OV2008) more sensitive to the DOC/DOX combination [41]. Synergy between DOC and anthracyclines was seen previously in *in vitro* studies [42–44]. DOC was synergistic when combined with DOX or epirubicin at dose levels equal to their IC50 values in PC3, DU145, and LnCap cells [43]. Despite promising results* in vitro*, DOC-based drug combinations need to be explored further. 

The major advantage of combination therapy is avoiding unnecessary toxicity through drug synergism. The combination effects of multiple drugs are frequently evaluated using the unified theory [45, 46]. According to the theory, synergy is concluded when the combined use of multiple drugs at specific dose levels leads to therapeutic efficacy equivalent to or greater than the sum of the antitumor effects achieved by each drug individually at the same dose levels. 

Although drug regimens are dependent on the dosing schedule [47], this study is focused on either single-drug treatments or multiple-drug treatments of DOC and DOX at various mixing ratios. Four combination settings are being tested by adjusting the ratios between DOC and DOX (DOC/DOX) that range between 10^−6^~10^8^. Moreover, the nature of the DOC/DOX combination is studied when the drug concentrations are fixed for both of them at their half maximal inhibitory concentrations (IC50). We expect that the optimal mixing ratios determined in this study would be used to propose not only a new therapeutic regimen, but also promising drug combination delivery systems. 

## 2. Materials and Methods

### 2.1. Materials

Docetaxel (DOC) and doxorubicin (DOX, hydrochloride salt) were purchased from LC Laboratories (Woburn, MA). Dimethyl sulfoxide (DMSO, molecular biology grade, ≥99.9%) and resazurin sodium salt were obtained from Sigma Aldrich (St Louis, MO). Sodium hydroxide (NaOH), sodium chloride (NaCl), and anhydrous sodium phosphate dibasic (Na_2_HPO_4_) were from VWR (West Chester, PA). Sodium phosphate monobasic dihydrate (NaH_2_PO_4_·2H_2_O) was obtained from Fisher Scientific (Fair Lawn, NJ). Phosphate-buffered saline (PBS, 0.1 M, pH 7.2, 0.9% NaCl) was prepared in house as a mixture of NaCl, Na_2_HPO_4_, and NaH_2_PO_4_·2H_2_O. The pH was adjusted to 7.2 with 1 M NaOH. 

### 2.2. Drug Formulations

DOC was dissolved in DMSO at 10 mg/mL (stock solution) and DOX stock solutions (10 mg/mL) were prepared by dissolving the drug in sterile deionized water (DI H_2_O). Stock solutions were stored in several aliquots at −20°C to avoid repetitive freeze-thaw cycles. DOC and DOX stock solutions were serially diluted using DMSO and DI H_2_O, respectively, to prepare drug working solutions. Working solutions were diluted 100 times with cell-culture media before treatment as described below.

### 2.3. Cell Lines

Human prostate cancer cell lines PC3 (prostate grade IV adenocarcinoma, CRL-1435) and DU145 (prostate carcinoma, HTB-81) were obtained from the American Type Culture Collection (ATCC, Manassas, VA). PC3 and DU145 were cultured in F12K (ATCC, Manassas, VA) and RPMI 1640 (Atlanta Biologicals, Lawrenceville, GA) media, respectively. The cell culture media were supplemented with 10% fetal bovine serum (FBS) in the presence of penicillin (100 U/mL) and streptomycin (100 *μ*g/mL). FBS, Penicillin, Streptomycin, and 0.25% trypsin/EDTA were from Gibco-Invitrogen Corporation (Carlsbad, CA). Cells were maintained in the logarithmic growth phase at 37°C in a humidified atmosphere containing 5% CO_2_ at all times. Cells were tested routinely for mycoplasma contamination. PC3 (5,000 cells/well) and DU145 (3,000 cells/well) were seeded in 96-well plates and allowed to adhere overnight prior to drug exposure. After preincubation, the cell culture media were replaced with drug-containing media. The cells were exposed to drugs for 72 hours, followed by cell viability assessment for single and combination drug treatments as described below.

### 2.4. Drug Treatment 

**Figure 1:**
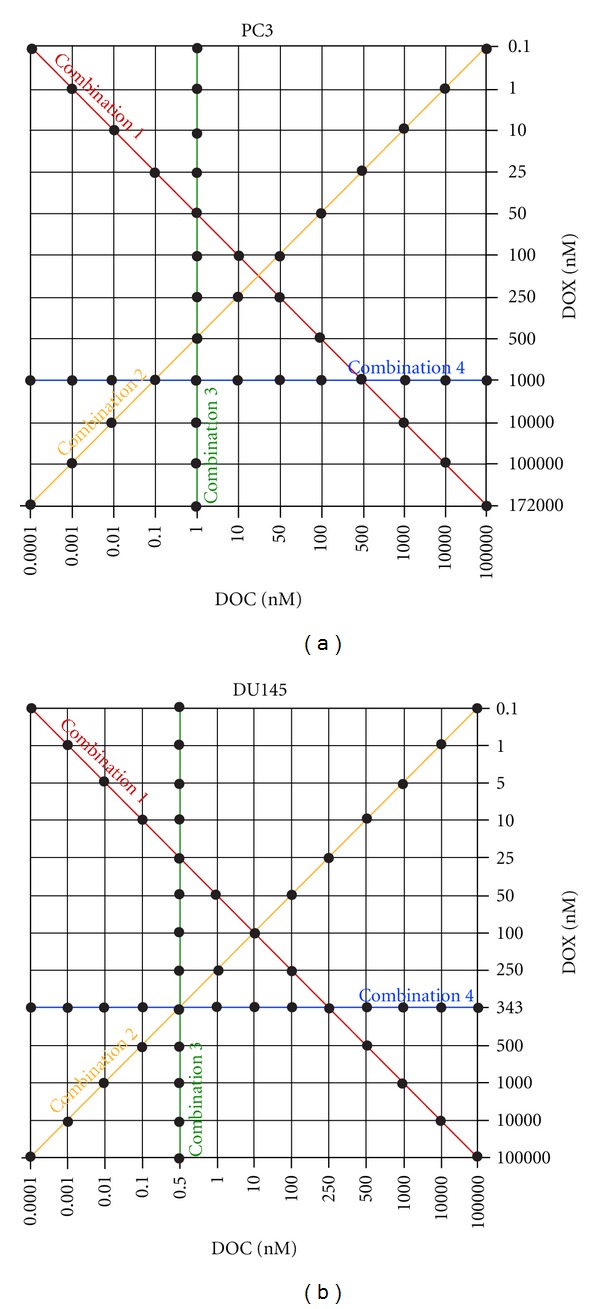
Drug combination settings used in this study. PC3 and DU145 cells were exposed to DOC and DOX at various concentrations and mixing ratios for 72 hours. Dots on the lines denoted as Combination 1, 2, 3, and 4 indicate data points used to determine the combination index and cytotoxicity values.

We first determined the half maximal inhibitory concentration (IC50) values of DOC and DOX for each cell line. Drug concentrations ranged from 10^−4^ to 10^5^ nM for the single-drug treatment. Multiple-drug treatment effects were then evaluated in four combination settings (Figure 1). Both DOC and DOX concentrations were variable in Combination 1 and Combination 2. Combination 3 and Combination 4 were designed to investigate combination effects of one drug when the IC50 value of the other drug remained fixed. In Combination 3, cells were treated at the fixed IC50 of DOC in combination with variable DOX concentrations. Cells in Combination 4 were exposed at the fixed IC50 dose level of DOX as DOC concentrations were variable. Combination effects of DOC and DOX were further investigated at equipotent concentrations of the IC50 values for each drug (7 × 7 combination).

### 2.5. Cell Viability Assay 

Cell viability was assessed using a resazurin assay that indicates mitochondrial metabolic activity in live cells [48, 49]. Briefly, 10 *μ*L of a 1 mM resazurin solution in PBS was added to the vehicle- and drug-treated cells at the end of the treatment period. Cell viability was determined three hours later by reading the fluorescence at 560 nm (Ex)/590 nm (Em). The fluorescence signals were quantified using a Spectramax M5 plate reader (Molecular Devices) equipped with a SoftMaxPro software.

### 2.6. Data Analysis 

GraphPad Prism (version 5.02 for Windows, GraphPad Software Inc., San Diego, USA) was employed to produce dose-response curves by performing nonlinear regression analysis. The viability of the treated cells was normalized to the viability of the untreated (control) cells, and cell viability fractions were plotted versus drug concentrations in the logarithmic scale. IC50 values were reported as mean values. The unified theory, introduced byChou[45, 46], was used to evaluate the synergy, additivity and antagonism of the DOC/ DOX combination in PC3 and DU145 cells. Combination index (CI) values were determined using the following equation:

(1)CI=[DOC][DOC]x+[DOX][DOX]x+[DOC]×[DOX][DOC]x×[DOX]x.

We used the mutually nonexclusive model, based on the assumption that drugs act through entirely different mechanisms of action. [DOC] and [DOX] are drug concentrations of DOC and DOX in combination, inhibiting *x*% of cell viability. [DOC]_*x*_ and [DOX]_*x*_ are the doses of DOC and DOX alone, respectively, inhibiting *x*% of cell viability. CI values were used to determine synergy (CI < 0.9), additivity (0.9 < CI < 1.1), and antagonism (CI > 1.1) of the drug combinations tested.

Experiments were conducted in triplicate (*n* = 3) with 6 replications at each drug concentration level (*r* = 6).

## 3. Results

### 3.1. Single-Drug Treatment

**Figure 2:**
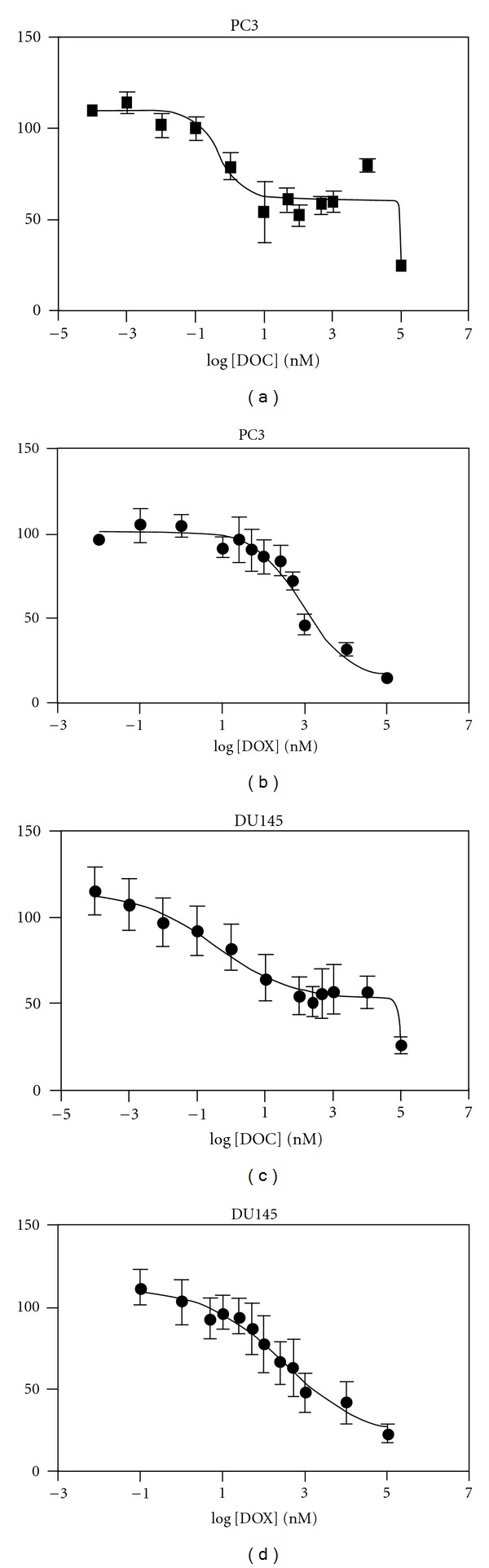
Dose response curves. PC3 and DU145 cells were exposed to either DOC (a) and (c) or DOX (b) and (d) for 72 hours, followed by the resazurin assay to determine cell viability (mean ± SD, *n* = 6).

IC50 values of DOC and DOX alone were determined in PC3 and DU145 as summarized in Table 1. DOC showed IC50 of 0.598 nM and 0.469 nM for PC3 and DU145, respectively. Dose-response curves in Figure 2, however, indicate that no complete cell death was seen in either of the cell lines treated with DOC up to 10^3^ nM (Figures 2(a) and 2(c)). Cell viability decreased significantly as DOC concentrations increased to 10^5^ nM. Biphasic dose-response curves revealed that the half effective dose (EC50) values of DOC between the first and second phase were significantly different (Table 1). Precise determination of the EC50 values of DOC was unsuccessful in the second phase (EC50 (2)) because we could not test concentrations higher than 10^5^ nM due to the precipitation of DOC in the cell culture media. The IC50 values of DOX were estimated equal to 908 nM and 343 nM for PC3 and DU145, respectively. Although the IC50 values of DOX were higher than those of DOC in both cell lines, DOX induced cell death in a dose-dependent manner described by a monophasic dose-response curve (Figures 2(b) and 2(d)). These results indicate that the potency of each drug cannot be compared directly simply by using the IC50 values. Therefore, we used DOC at a dose level equal to the EC50 (1) value when combined with DOX. Overall cytotoxicity trends show that DU145 is more sensitive to DOC and DOX single-drug treatments than PC3. 

### 3.2. Synergy of Multiple Drug Combinations

**Figure 3:**
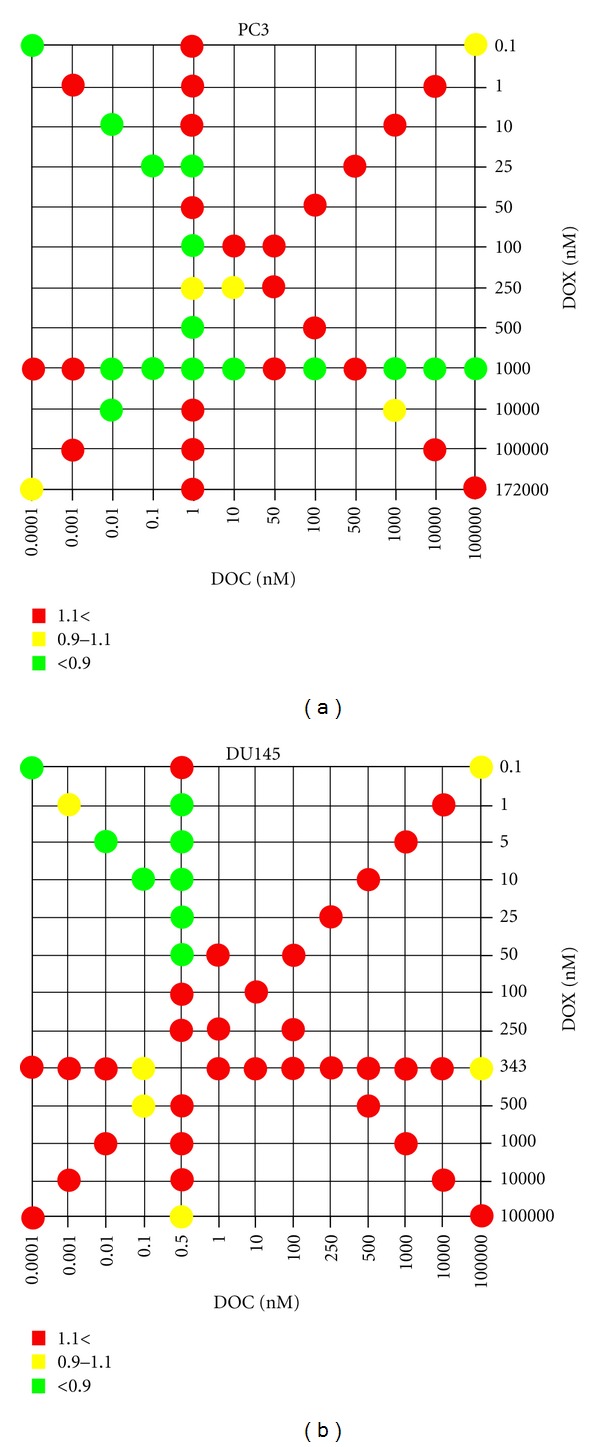
Synergy maps compiling combination index (CI) values. The dots in the figure indicate antagonism (CI > 1.1, red), additivity (0.9 < CI < 1.1, yellow), and synergism (CI < 0.9, green) between DOC and DOX against PC3 and DU145 cells at combination settings shown in Figure 1. Experiments were performed in triplicate.

**Figure 4:**
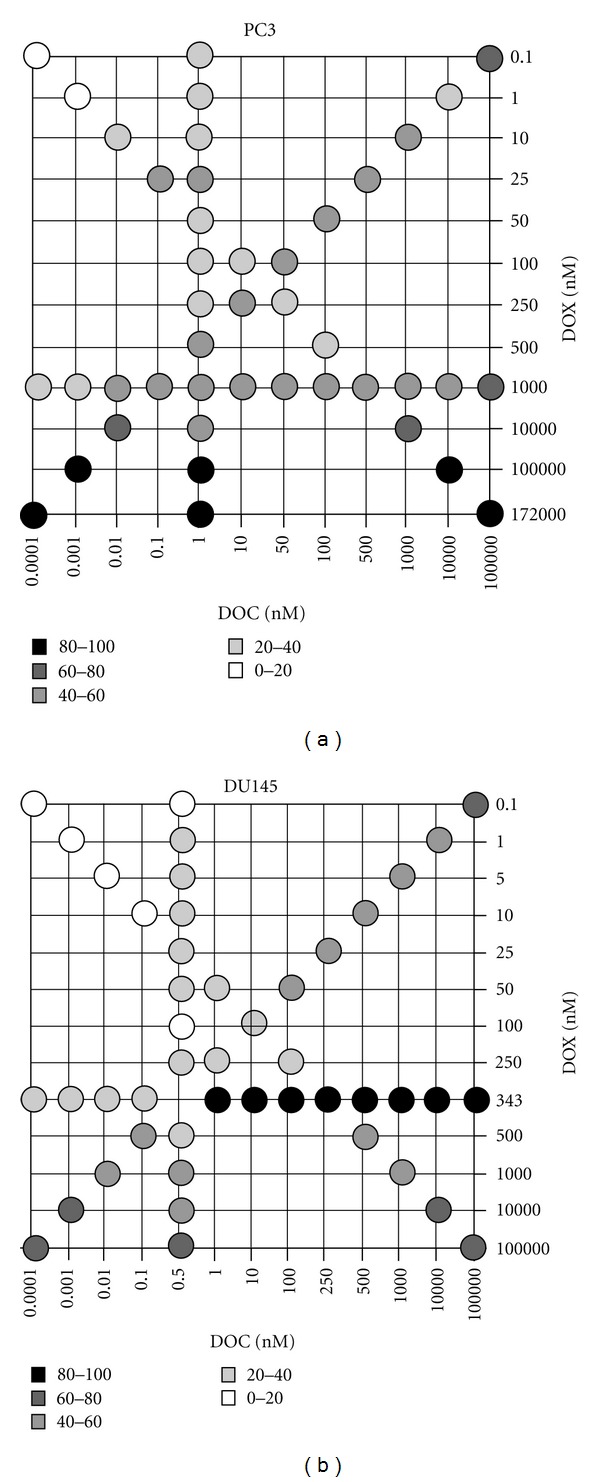
Drug combination cytotoxicity. The grayscale dots in the figure show average percent cell death of PC3 and DU145 cells at each combination setting from triplicate experiments shown in Figure 3.

Figure 3 shows the synergy maps of the DOC and DOX combination, and Figure 4 represents the cytotoxicity of the drug combinations for PC3 and DU145.

Drug synergy and significant cell death (~60%) was seen in PC3 at 10^4^ nM DOC and 10^3^ nM DOX. However, the DOC concentration was too high to take advantage of the drug combination in terms of achieving our goal of obtaining the desirable therapeutic effect by administering low doses of the drugs in combination. We observed moderate cytotoxicity on the PC3 (49~58% cell death) at the concentration levels of DOC and DOX equal to their IC50 values. Moreover, drug synergy was observed when PC3 cells were treated with low doses of DOC in combination with DOX doses approximating its IC50 values. Drug synergy was accompanied by effective cell death (>50%) at DOC levels varying from 0.01 to 10 nM, while the DOX dose was equal to its IC50 value. 

DOC/DOX combinations in DU145 appeared additive or antagonistic in most combination settings (Figure 3). However, synergy was observed in the DU145 at the highly limited concentration range of 0.5 nM DOC plus DOX between 1–50 nM. Cell death reported on the DU145 was 11~33% for the synergistic combinations (Figure 4). Interestingly, significant cell death was noted when doses of DOC ranging from 1 to 10^4^ nM combined with 343 nM of DOX (>80%) (Figure 4). Nevertheless, all those mixing ratios proved to be antagonistic (Figure 3). 

These results demonstrated that the DOC/ DOX combination was synergistic in PC3, while synergy was seen in limited concentration levels in the DU145. 

### 3.3. Synergy of the 7 × 7 Combination

**Figure 5:**
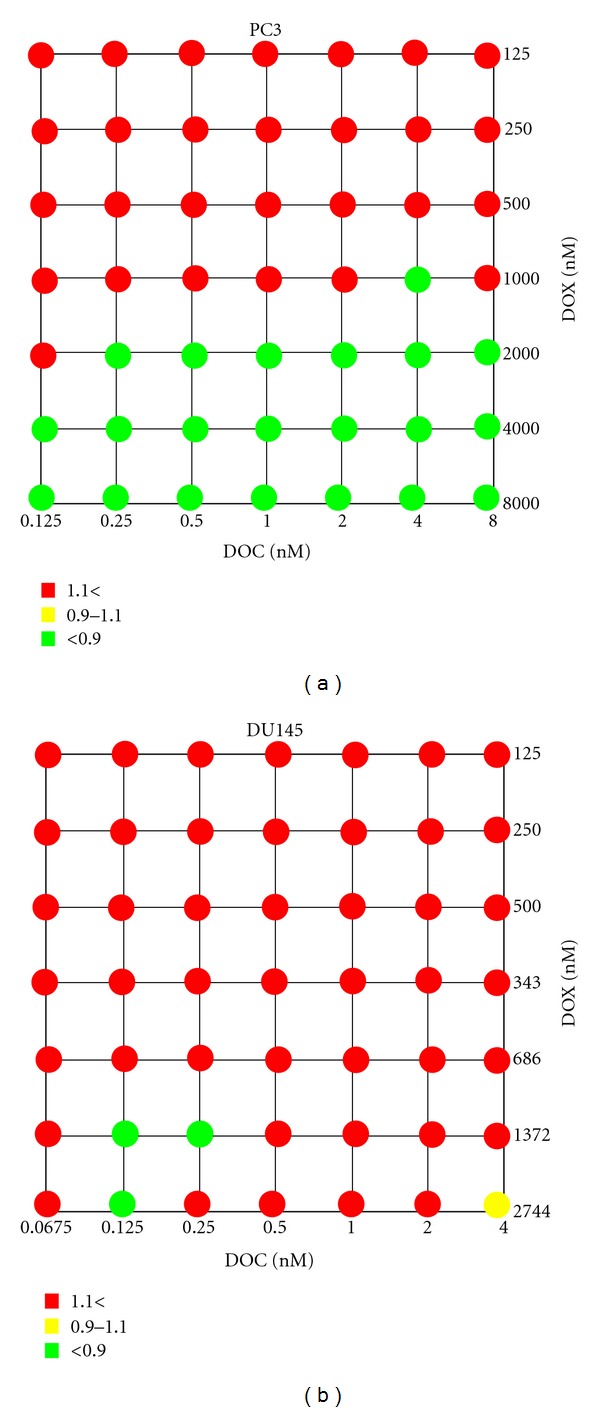
Focused synergy maps compiling combination index (CI) values. The CI values were determined with a narrower dose area with 7 × 7 drug combination design. Drug antagonism (red), additivity (yellow), and synergism (green) were determined for PC3 and DU145 cells exposed to DOC and DOX for 72 hours.

**Figure 6:**
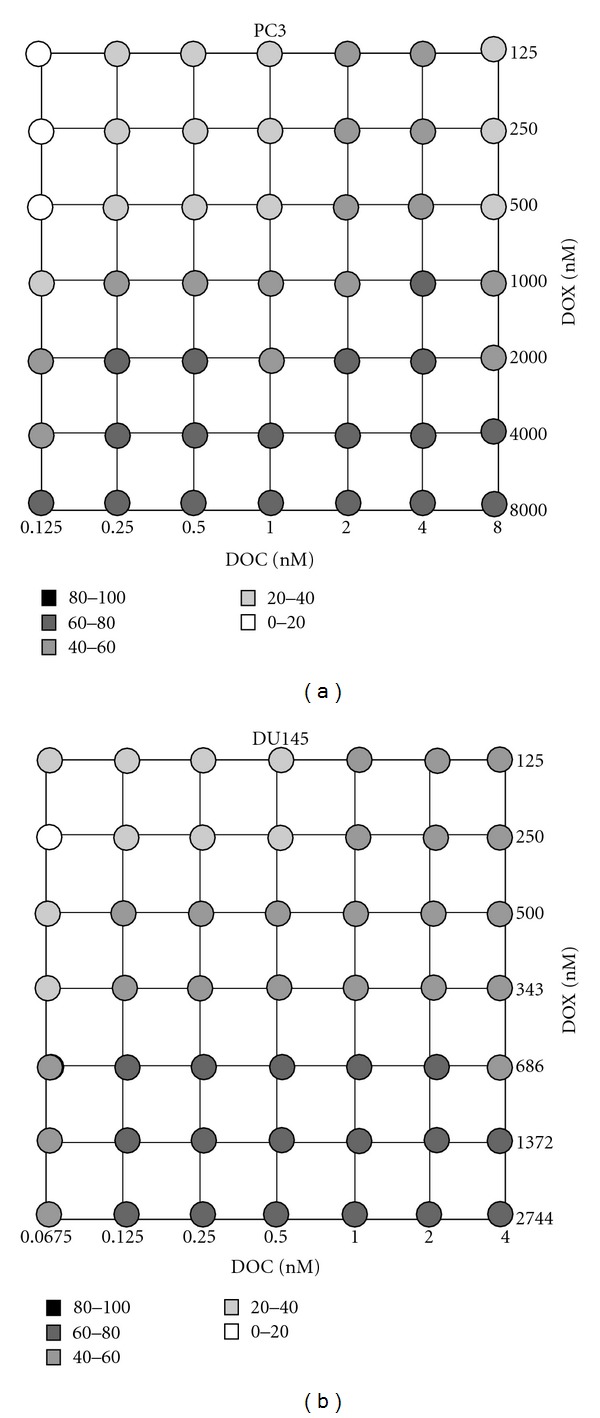
Focused drug combination cytotoxicity. The grayscale dots in the figure demonstrate percent cell death of PC3 and DU145 with 7 × 7  DOC/DOX combination settings.

We further investigated DOC/ DOX combination effects by exposing the prostate cancer cells to DOC/ DOX mixing ratios at seven dose levels (7 × 7 combination) (Figures 5 and 6). These drug concentrations were selected based on predetermined IC50 values for each drug (data not shown).  Figure 5 shows the synergy maps, indicating that the DOC/ DOX combination effects can be controlled by simply changing the mixing ratios of two drugs at their IC50 values. PC3 were sensitive to the combination of low doses of DOC varying from 0.125 to 0.5 nM (25~100% of DOC IC50) with 2,000~8,000 nM DOX (2~8 fold of DOX IC50). In DU145, the DOC/ DOX combination showed strong synergy only when 0.125 or 0.25 nM DOC was combined with 1,372 nM DOX (4-fold DOX IC50) (Figure 5). Although DOX appeared to make prostate cancer cells more susceptible to DOC, by decreasing the DOC doses necessary for cell death, the observed cell death never exceeded 80% in either of the cell lines used. Data collected regarding the combination index values and the cytotoxicity presented on Figures 5 and 6, respectively, also suggests that in the DU145 cell line, the DOC/DOX combination might have a narrow mixing ratio window for exerting its antitumor activity effectively. 

## 4. Discussion

### 4.1. DOC/DOX Combination in Prostate Cancer *In Vitro* Models

Prostate cancers that no longer respond to hormone therapy are categorized as hormone-refractory and are typically accompanied by metastatic lesions. DOC is one of the most potent anticancer drugs for HRPC treatment [27, 28], yet its clinical applications are limited due to low bioavailability and high toxicity. DOX is widely used clinically for the treatment of various cancers and has been used in the past in prostate cancer patients [31, 50–53]. In this study, we investigated the cytotoxic effect of the DOC/ DOX combination on metastatic prostate cancer cells derived from the bone (PC3) and brain (DU145).

A number of studies [43, 47], including our own, have shown that DOX causes dose-dependent cytotoxicity in cancer cells. We also confirmed that the cytotoxic profile of DOX is a monophasic one on both cancer cell lines. In respect to DOC, our data are in agreement with previous findings that showed biphasic cellular response of breast cancer cell lines to DOC [54]. At low dose levels, DOC is known to cause nonapoptotic cell death, which is accompanied by senescence, necrosis, and mitotic catastrophe [54–57]. Mitotic catastrophe is cell death occurring during metaphase and distinct from apoptosis-related cell death observed at DOC concentrations at the micromolar range. Therefore, we hypothesize that the initial decrease on the observed cell viability (EC50 (1)) is due to mitotic catastrophe, while apoptosis is the cause for the cell death observed at DOC concentrations higher than 10 *μ*M (Figure 2). Interestingly, pharmacokinetic studies have shown that the recommended administration schedule of DOC results in drug blood levels in the micromolar range [58, 59]. Therefore, it is safe to attribute the observed antitumor activity of DOC in the clinic to DOC-induced cell apoptosis.

DOC and DOX cause cytotoxic effects through completely different mechanisms of action. DOC and other taxanes act by binding to microtubules and change the formation of the cell cytoskeleton and cell signaling [15]. Furthermore, DOC treatment leads to phosphorylation of the antiapoptotic protein Bcl2 [16], preventing the protein from dimerizing with its proapoptotic partner Bax. The readily available Bax forms homodimers instead and induces apoptosis. Topoisomerase II inhibitors, including DOX, primarily induce cell death through DNA damage [17, 19]. They are also known to induce free-radical DNA injury and lipid peroxidation [60, 61]. Additionally, the interaction between anthracyclines and the cytoskeleton has been studied [62]. DOX is known to delay G-actin polymerization and prevents the elongation of small actin filaments performed during the polymerization* in vitro* [63]. Therefore, cells in dynamic movement or mitosis could be more susceptible to DOX treatment. G-actin rearrangement failed in breast cancer cells treated with cytochalasin B, a microtubule inhibitor, being in combination with DOX [64]. These results suggest that the mechanisms of action of DOC and DOX are not distinctively independent and that actin could be a potential target that can be inhibited cooperatively by DOC and DOX in drug combinations tested in this study.

### 4.2. Clinical Impact of the Multiple Drug Combination

The antitumor activity of a cytotoxic agent depends on the drug levels at the tumor site. The presence of the anticancer agent at an optimal concentration is essential for interaction with its target and for inducing a pharmacodynamic effect. Moreover, intracellular metabolism, interaction with transporters, and interactions with concomitantly given anticancer agents may have an impact on the pharmacokinetic profile of a cytotoxic agent. For these reasons, we investigated the cellular response of PC3 and DU145 to the DOC/ DOX combination by varying the concentrations and mixing ratios of the drugs (Figure 1). In Figure 3, we calculated the combination index (CI) values of DOC/ DOX combination and determined synergism, additivity, and antagonism. The effect of the DOC/ DOX combination on the cell viability is presented on Figure 4. The information collected by both the synergy maps and the DOC/ DOX-induced cell death for the various drug mixing ratios provided us with a more thorough understanding of the potential *in vivo* cytotoxic effect of the aforementioned drug combination. It was revealed that cell death at high drug concentrations was not always attributed to effective drug combinations. In most cases, drug antagonism was observed at dose levels greater than the IC50 values of each drug. In PC3, strong drug synergism and effective cell death (>50%) were seen in concentration ranges close to the IC50 value of each of the drugs. Interestingly, the DU145 cell line was more sensitive to DOC or DOX alone but showed greater cell survival when the two drugs were combined. Further studies are necessary to elucidate the cellular mechanisms behind the limited cellular response of DU145 when treated with the DOC/ DOX combination. 

### 4.3. Clinical Significance of the Synergy Observed with the 7 × 7 Combination

The DOC/ DOX combination was previously tested in breast cancer* in vitro* models, using 5 nM DOC and 100 nM DOX [41]. In our study, the nature of the DOC/ DOX combination was assessed in more detail at various mixing ratios of DOC and DOX concentrations equal to their IC50 values. Synergy was seen in a broad drug concentration range in PC3, while only limited combination settings induced synergistic effects in DU145 (Figure 5).  Figure 6 indicates that cell death of potential clinical significance was observed at dose levels greater than the IC50 values for DOX. This finding is more pronounced in the PC3 cell line. 

We found that DOC can be potent in prostate cancer at nanomolar concentrations by using DOX as a combination counterpart, although the anthracycline is 1000 times less toxic than DOC. We were able to identify fewer cases of synergy in the DU145 than in the PC3 cell line. We observed strong synergy at DOC dose levels lower than its IC50 value plus DOX at dose levels 2–4-fold higher than its IC50 in both PC3 and DU145, suggesting that the significantly low DOC concentrations would be sufficient to trigger the synergistic effects of the drug combination. These results are important because concentrations equal to the IC50 values for each cytotoxic agent can be achieved in the clinical setting [58, 59, 65, 66]. However, the efficacy of the drug combination represented as cell death remained lower than 75%, indicating that DOC/ DOX combinations are moderately cytotoxic. To conclude, even though complete cell death was not seen in our studies, the DOC/ DOX combination could potentially benefit prostate cancer patients by reducing the DOC-mediated toxicity. We expect that the therapeutic efficacy of the proposed DOC/ DOX combination will be enhanced by further optimization of the regimen.

## 5. Conclusions

In this study, we identified optimal combination settings for DOC and DOX, anticancer drugs commonly used in clinical applications, in hormone-refractory prostate cancer cells (PC3 and DU145). Strong drug synergy was seen at dose levels lower than the IC50 values of DOC in combination with 2~4-fold IC50 values of DOX. In comparison to existing cytotoxicity assays that evaluate drug potency simply by using IC50 values, the synergy maps allowed facile and reasonable evaluation of the drug combination effect, irrespective of the monophasic or biphasic cytotoxicity patterns of drugs used in combination. By combining information that includes the combination index values and the cytotoxicity observed, we were able to identify drug ratios of DOC and DOX that could be potentially worth testing in future *in vivo* studies. We also found that drug synergy was not always correlated with cell death when a drug with high potency (DOC) was used at dose levels higher than its IC50 in combination with a less potent drug (DOX). Our study demonstrated optimal concentration ratios of DOC and DOX in combination for the treatment of PC3 and DU145, which could improve chemotherapy for prostate cancer patients and can be used for the design of a multiple-drug delivery system in the future. In conclusion, the combination of DOC with DOX evaluated in this study potentiates the cytotoxic properties of DOC given at significantly reduced doses compared to the clinically relevant ones, and thus could potentially protect cancer patients from unnecessary DOC-related toxicity.

## Figures and Tables

**Table 1:** Cytotoxicity of docetaxel (DOC) and doxorubicin (DOX) in hormone-refractory PC3 and DU145 cancer cell lines^a^. IC50 values are given as mean.

	PC3			DU145		
	IC50^b^	EC50 (1)^c^	EC50 (2)^c^	IC50^b^	EC50 (1)^c^	EC50 (2)^c^
DOC (nM)	0.598	0.500^d^	92,000^d^	0.469	0.392	104,000^d^
DOX (nM)	908	N.D.	N.D.	343	N.D.	N.D.

^a^Cells were treated with either DOC or DOX alone.

^b^IC50 denotes the half maximal inhibitory concentration.

^c^EC50 (1) and EC50 (2) indicate the half effective concentrations in the first (1) and second (2) phase of the biphasic curve used to fit the cell viability data.

^d^Approximate estimation due to insufficient data points.

N.D: Not determined.
